# DermaCalibra: A Robust and Explainable Multimodal Framework for Skin Lesion Diagnosis via Bayesian Uncertainty and Dynamic Modulation

**DOI:** 10.3390/diagnostics16040630

**Published:** 2026-02-21

**Authors:** Ben Wang, Qingjun Niu, Chengying She, Jialu Zhang, Wei Gao, Lizhuang Liu

**Affiliations:** 1 Shanghai Advanced Research Institute, Chinese Academy of Sciences, Shanghai 201210, China; wangben2024@sari.ac.cn (B.W.); niuqj@sari.ac.cn (Q.N.); shechengying2024@sari.ac.cn (C.S.); zhangjl@sari.ac.cn (J.Z.); 2 University of Chinese Academy of Sciences, Beijing 100049, China; 3 Department of Dermatology, The Second People’s Hospital of Changzhou, Changzhou 213003, China

**Keywords:** skin lesion diagnosis, multimodal learning, deep learning, class imbalance, interpretability, computer-aided diagnosis, uncertainty estimation

## Abstract

**Background:** Accurate and timely diagnosis of skin lesions, including Melanoma (MEL), Basal Cell Carcinoma (BCC), Squamous Cell Carcinoma (SCC), Actinic Keratosis (ACK), Seborrheic Keratosis (SEK), and Nevus (NEV), is often hindered by the severe class imbalance and high morphological similarity among pathologies in clinical practice. Although multimodal learning has shown potential in resolving these issues, existing approaches often fail to address predictive uncertainty or effectively integrate heterogeneous clinical metadata. Therefore, this study proposes DermaCalibra, a robust and explainable multimodal framework optimized for small-scale, imbalanced clinical datasets. **Methods:** The proposed framework integrates three essential modules: First, the Attention-Based Multimodal Channel Recalibration (AMCR) module introduces a probabilistic Bayesian uncertainty estimation mechanism via Monte Carlo dropout to adjust focal loss weights, prioritizing features from underrepresented classes. Second, the Metadata-Driven Dynamic Feature Modulation and Cross-Attention Fusion (MDFM-CAF) module, designed to resolve inter-class visual ambiguity, dynamically rescales dermoscopic feature maps using non-linear clinical context transformations. Lastly, the Gradient Feature Attribution (GFA) module is implemented to provide pixel-level diagnostic heatmaps and metadata importance scores. **Results:** Evaluated on the PAD-UFES-20 dataset, DermaCalibra achieves a balanced accuracy (BACC) of 84.2%, outperforming current state-of-the-art (SOTA) methods by 3.6%, and a Macro Area Under the Receiver Operating Characteristic Curve (Macro AUC) of 96.9%. Extensive external validation on unseen hospital and synthetic datasets confirms robust generalizability across diverse clinical settings without the need for retraining. **Conclusions:** DermaCalibra effectively bridges the gap between deep learning complexity and clinical intuition through uncertainty-aware reasoning and transparent interpretability. The framework provides a reliable and scalable computer-aided diagnostic tool for early skin lesion detection, particularly in resource-limited clinical environments.

## 1. Introduction

**Figure 1:**
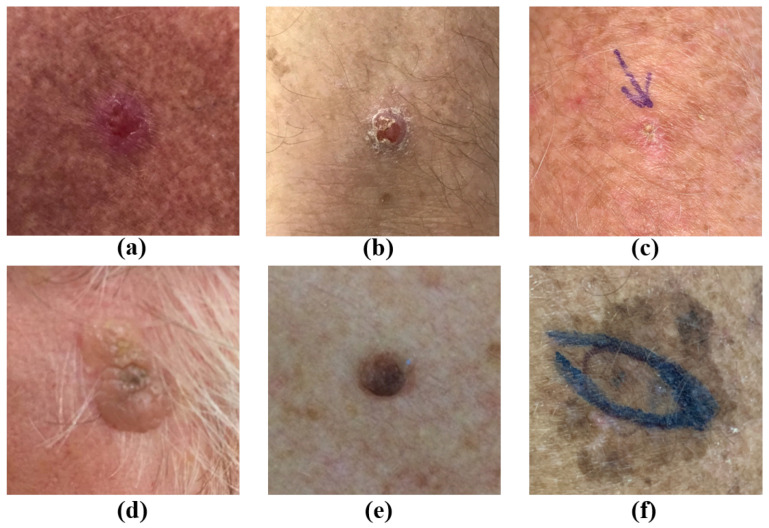
Dermoscopic images from PAD-UFES-20 showing six skin lesion classes: (**a**) BCC (Basal Cell Carcinoma); (**b**) SCC (Squamous Cell Carcinoma); (**c**) ACK (Actinic Keratosis); (**d**) SEK (Seborrheic Keratosis); (**e**) NEV (Nevus); (**f**) MEL (Melanoma).

Skin cancers such as Melanoma (MEL) and Basal Cell Carcinoma (BCC) are a growing global health concern, and their rising incidence rates combined with late detection often lead to severe outcomes [1,2,3,4,5], meaning that early diagnosis is vital for effective treatment. Accurate identification is particularly critical to clinical prognosis for malignant types such as Melanoma (MEL), Basal Cell Carcinoma (BCC), and Squamous Cell Carcinoma (SCC); however, the diagnostic process faces significant challenges, the largest of which is asymmetry. Indeed, in clinical dermatology, the ABCD rule explicitly defines asymmetry as the most critical diagnostic criterion for differentiating malignant from benign lesions. In automated diagnostic systems, this challenge manifests as asymmetry in data distribution (severe class imbalance) and asymmetry in information content (disparity between high-dimensional dermoscopic images and low-dimensional patient metadata). Another challenge is that some conditions, for example, BCC and SCC, present as visually similar (Figure 1). Furthermore, in datasets like PAD-UFES-20, rare conditions like Actinic Keratosis (ACK) and SCC are underrepresented [6].

These issues are particularly critical in resource-limited areas, such as rural regions, where dermatological expertise and advanced imaging are scarce, delaying diagnosis and worsening outcomes [7]. Automated, interpretable AI systems can bridge this gap.

Deep learning has advanced skin lesion classification using dermoscopic images [8], yet image-only models often overlook the clinical context, such as patient demographics and lesion history, which dermatologists rely on for definitive diagnosis. Multimodal approaches aim to mimic this expert diagnostic process by integrating images with metadata. For instance, the approach of Pacheco and Krohling employs attention-based fusion [9], while that of Li et al. utilizes multiplication-based strategies [10]. Recent advancements include super-resolution frameworks [11], Bayesian networks [12], and multimodal large language model approaches [13]. However, despite this progress, existing methods frequently struggle with the severe class imbalance inherent in clinical datasets like PAD-UFES-20, fail to provide robust support for rare pathologies, and offer limited interpretability [14,15].

The clinical imperative for enhanced multimodal and uncertainty-aware approaches is further substantiated by recent advancements in biophotonics. Emerging imaging modalities, particularly hyperspectral imaging (HSI), have shown significant potential in capturing spectral signatures beyond traditional RGB dermoscopy to enhance skin cancer classification [16]. While HSI provides superior diagnostic granularity, its need for specialized hardware and large-scale datasets often restricts its accessibility in standard clinical workflows [17]. This necessitates timely and complementary research focusing on enhancing the robustness and uncertainty-awareness of diagnostics using standard clinical and smartphone-based imaging, particularly in practical settings where data is limited and imbalanced.

The choice of architectural backbone also plays a critical role in clinical applicability. Vision Transformer (ViT)-based models have demonstrated promise by capturing global lesion features through attention mechanisms, reporting high accuracy on large benchmarks [18,19,20]. However, ViTs generally demand vast amounts of training data and computational resources, rendering them prone to overfitting on smaller clinical datasets. In contrast, ResNet50-based approaches exhibit greater robustness on small-scale datasets like PAD-UFES-20 [21].

Overall, existing multimodal or uncertainty-aware models that are not constrained by architectural complexity often fail to adequately address the noise and imbalance found in practical clinical settings, as they typically treat uncertainty as a post hoc evaluation metric rather than a dynamic training driver. Thus, there remains an unaddressed need for a framework that fundamentally differs from existing architectures by resolving these issues without sacrificing transparency or efficiency.

To bridge these gaps, we propose DermaCalibra, a novel multimodal framework that replaces brute-force architectural scaling with an uncertainty-guided recalibration strategy. Built upon a ResNet50 backbone, it is specifically optimized for data-limited and imbalanced environments. DermaCalibra introduces three innovative modules to resolve clinical diagnostic bottlenecks: (1) the Attention-Based Multimodal Channel Recalibration (AMCR) module, which leverages Bayesian uncertainty estimation via Monte Carlo dropout to dynamically adjust focal loss weights, ensuring the model prioritizes underrepresented malignant classes like SCC; (2) the Metadata-Driven Dynamic Feature Modulation and Cross-Attention Fusion (MDFM-CAF) module, which effectively merges dermoscopic features with non-linear clinical context transformations to resolve inter-class visual ambiguities; and (3) the Gradient Feature Attribution (GFA) module, which provides pixel-level diagnostic heatmaps and metadata importance scores to foster clinical trust.

Unlike complexity-unconstrained models, DermaCalibra provides superior efficiency and interpretability, rendering it well-suited for resource-constrained clinical deployment. Evaluated on PAD-UFES-20, it achieves a BACC of 84.2%, outperforming state-of-the-art (SOTA) methods by 3.6%. Through extensive external validation and a discussion of real-world deployment challenges, including inference duration and ethical ramifications, we demonstrate a robust and scalable solution for early skin lesion diagnosis.

Our primary contributions can be summarized as follows:

1. AMCR for robust class imbalance handling: Leveraging Monte Carlo dropout, AMCR applies Bayesian uncertainty estimation to dynamically adjust focal loss weights, improving recognition of rare malignant classes and boosting BACC by 3.6%.
2. MDFM-CAF for enhanced multimodal integration: By combining dynamic convolution with cross-attention, MDFM-CAF effectively fuses image and metadata features, enabling precise discrimination of visually similar lesions like BCC and SCC.
3. GFA for clinical interpretability: GFA generates detailed heatmaps and metadata scores, highlighting critical lesion features and clinical factors, thereby supporting transparent and trustworthy dermatological assessments.
4. SOTA performance and scalability: Achieving 84.2% BACC and 96.9% Macro AUC on PAD-UFES-20, DermaCalibra outperforms existing approaches and facilitates reliable diagnostics in resource-limited settings.

## 2. Methods

**Figure 2:**
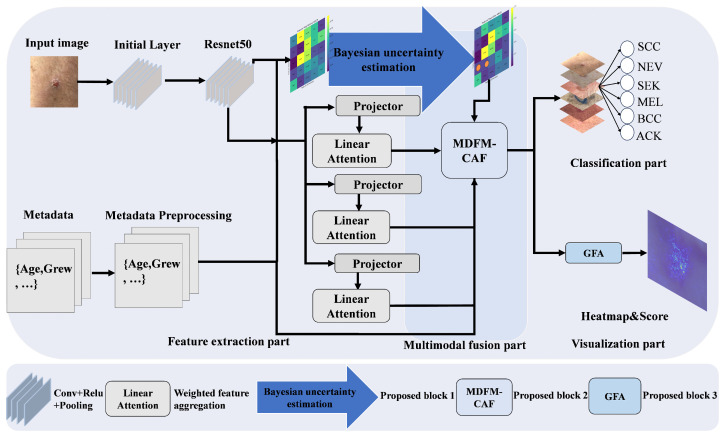
Architecture of the DermaCalibra model, illustrating image feature processing through ResNet50, metadata embedding, AMCR for channel recalibration with uncertainty estimation, MDFM-CAF for dynamic modulation and cross-attention fusion, GFA for heatmaps and metadata scores, and classification of six skin lesion classes.

The DermaCalibra framework is built on a ResNet50 backbone [22] due to its proven robustness on small datasets like PAD-UFES-20 (2298 images), balancing computational efficiency (23.6 M parameters) and performance. This choice is supported by Pundhir et al. [23], who demonstrated that ResNet50, used in their model, outperforms other architectures on PAD-UFES-20 for skin lesion classification. Compared to modern architectures like Inception [24], DenseNet [25], and EfficientNet [26], used in related works [27], ResNet50 achieves superior performance with a lower risk of overfitting. Our model further integrates three key modules (AMCR, MDFM-CAF, GFA) to address class imbalance, low inter-class variability, and the need for interpretability in skin lesion diagnosis for the PAD-UFES-20 dataset. Below, we detail each module’s functionality, mathematical principles, dataset preprocessing, training procedures, and implementation details. Figure 2 illustrates the overall network architecture.

### 2.1. Attention-Based Multimodal Channel Recalibration (AMCR)

AMCR serves as the foundational innovation of *DermaCalibra*, designed to address the critical challenge of class imbalance in the PAD-UFES-20 dataset—particularly for rare skin lesions such as SCC—while enhancing feature differentiation for visually similar lesions like BCC and SCC. This module leverages Bayesian uncertainty estimation via Monte Carlo dropout [28,29] to tailor the model to the specific needs of skin lesion diagnosis. Given an input feature map $F\in R^{C\times H\times W}$ extracted from ResNet50’s layer4 (where C=2048, H=W=7) and a metadata vector $M\in R^{81}$ (81 dimensions, encompassing patient demographics and clinical features from PAD-UFES-20), AMCR computes a channel-wise attention vector through a mathematically rigorous process. Initially, *F* undergoes spatial aggregation via global average pooling to derive a channel descriptor $z\in R^{C}$:

(1) $$z_{c}=\frac{1}{H\times W}\sum_{i=1}^{H}\sum_{j=1}^{W}F_{c}\left(i,j\right),\;c=1,\ldots ,C$$

Concurrently, the metadata *M* is transformed through a fully connected layer, incorporating batch normalization, ReLU activation, and a high dropout rate (p=0.5). This structure serves as a dedicated regularization mechanism to mitigate adverse biases and prevent the model from disproportionately relying on individual metadata attributes:

(2) $$\begin{array}{c} e_{M}=\mathrm{Dropout}\left(\mathrm{ReLU}\left(\mathrm{BN}\left(W_{M}\cdot M+b_{M}\right)\right)\right),W_{M}\in R^{d\times 81},\;b_{M}\in R^{d} \end{array}$$

The channel descriptor *z* and metadata embedding $e_{M}$ are concatenated into a joint feature vector $\left[z;e_{M}\right]\in R^{C+d}$, which is then processed by a multilayer perceptron (MLP) with a hidden layer of size $C^{\prime }=256$, followed by a sigmoid activation to generate channel-wise attention weights $\alpha \in R^{C}$:

(3) $$\alpha =\sigma \left(W_{2}\cdot \mathrm{ReLU}\left(W_{1}\cdot \left[z;e_{M}\right]+b_{1}\right)+b_{2}\right),\;W_{1}\in R^{C^{\prime }\times \left(C+d\right)},\;W_{2}\in R^{C\times C^{\prime }}$$

where σ denotes the sigmoid function. The recalibrated feature map $F^{\prime }$ is then computed via element-wise multiplication of the attention weights with the original feature map:

(4) $$F_{c}^{\prime }=\alpha_{c}\cdot F_{c},\;c=1,\ldots ,C$$

The mathematical core of the AMCR lies in its Bayesian uncertainty estimation, which is pivotal for skin lesion diagnosis, where class imbalance can skew model performance. By applying Monte Carlo dropout with a dropout probability p=0.5 during training, the AMCR performs T=10 forward passes to sample a distribution of attention weights. The predictive uncertainty is quantified as the variance of the softmax outputs $\sigma^{2}\left(S_{c}\right)$ across these passes, which dynamically adjusts the focal loss weights $w_{c}$ to prioritize underrepresented classes:

(5) $$w_{c}=\frac{1}{1+\exp(-\lambda \cdot \sigma^{2}\left(S_{c}\right))},\;\lambda =0.1$$

**Figure 3:**
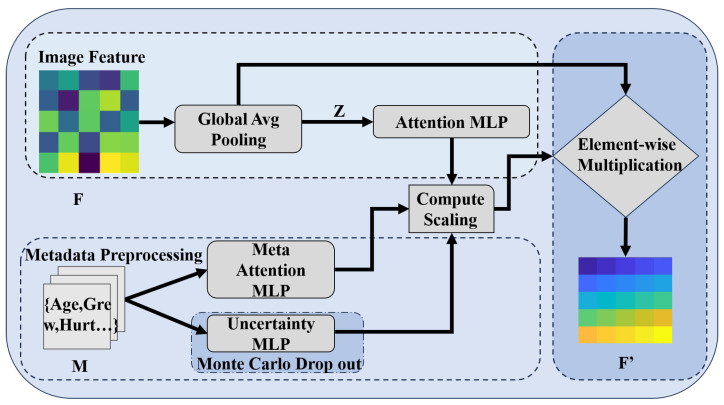
AMCR structure, illustrating metadata-driven channel recalculation with Bayesian uncertainty estimation to address class imbalance in skin lesion diagnosis.

This uncertainty-weighted loss function enhances the model’s sensitivity to rare lesions by amplifying the contribution of samples with high predictive variance, a critical adaptation for detecting SCC amidst dominant classes like NEV [30]. The Bayesian approach further enables a probabilistic interpretation of model confidence, aligning with the clinical need for reliable diagnostic thresholds in dermatology. AMCR introduces 0.3 M parameters to the model (total 23.6 M), with recalibration scaling factors α saved every 10 epochs to analyze metadata-driven channel importance, offering insights into the features most relevant to lesion classification. The module’s structure is depicted in Figure 3.

### 2.2. Metadata-Driven Dynamic Feature Modulation and Cross-Attention Fusion (MDFM-CAF)

MDFM-CAF constitutes a pivotal enhancement in DermaCalibra, dynamically integrating image features with metadata to overcome the shortcomings of static concatenation or multiplication-based fusion approaches [31]. Tailored to the challenges of skin lesion classification, this module addresses the critical need to differentiate visually similar lesions—such as BCC and SCC in the PAD-UFES-20 dataset—through a two-stage process comprising dynamic feature modulation and cross-attention fusion, optimizing metadata-driven feature representation for clinical precision [32].

To handle the inherent incompleteness of clinical data, we apply median imputation for numerical features and an “unknown” label for categorical ones. The MDFM-CAF module addresses potential metadata conflicts through dynamic modulation; by learning non-linear transformations γ and β, the model adaptively weighs clinical cues against dermoscopic features. This ensures that, in scenarios with missing or erroneous metadata, the framework gracefully transitions to image-heavy inference without a total loss of diagnostic utility.

In the dynamic modulation phase, the metadata embedding $e_{M}\in R^{512}$, derived from the AMCR module, informs the computation of modulation parameters $\gamma ,\beta \in R^{2048}$ via linear transformations. These parameters facilitate adaptive scaling and shifting of the recalibrated feature map $F^{\prime }\in R^{2048\times 7\times 7}$, enabling the model to emphasize channels informed by metadata attributes—such as patient age or lesion history—crucial for resolving inter-class variability between BCC and SCC:

(6) $$\gamma =W_{\gamma }\cdot e_{M}+b_{\gamma },\;\beta =W_{\beta }\cdot e_{M}+b_{\beta },\;W_{\gamma },W_{\beta }\in R^{2048\times 512}$$

The modulated feature map $F^{\prime \prime }$ is subsequently derived through a channel-wise affine transformation:

(7) $$F_{c}^{\prime \prime }=\gamma_{c}\cdot F_{c}^{\prime }+\beta_{c},\;c=1,\ldots ,2048$$

This modulation enhances the salience of dermoscopic features correlated with metadata, bolstering the model’s discriminative power across lesion classes. To ensure that this metadata-driven modulation does not introduce unfavorable biases, the GFA module is utilized to monitor feature importance scores across training epochs, allowing us to verify that the model maintains a balanced reliance on both visual hallmarks and clinical context rather than being biased by a few dominant demographic factors.

The cross-attention fusion stage refines this integration by projecting $F^{\prime \prime }$ into query (*Q*), key (*K*), and value (*V*) matrices via linear transformations, while $e_{M}$ is mapped to a metadata key $K_{M}$. This process merges spatial and contextual information through an advanced attention mechanism [33]:

(8) $$Q=W_{Q}\cdot F^{\prime \prime },\;K=W_{K}\cdot F^{\prime \prime },\;V=W_{V}\cdot F^{\prime \prime },\;K_{M}=W_{K_{M}}\cdot e_{M}$$

where $W_{Q},W_{K},W_{V}\in R^{2048\times 2048}$ and $W_{K_{M}}\in R^{2048\times 512}$. The cross-attention output *A* is computed using a scaled dot-product attention mechanism, guided by the metadata key to prioritize clinically relevant features:

(9) $$A=\mathrm{softmax}\left(\frac{Q\cdot (K^{T}+K_{M}^{T})}{\sqrt{2048}}\right)\cdot V$$

A residual connection then yields the fused feature map $F_{\mathrm{fused}}$:

(10) $$F_{\mathrm{fused}}=F^{\prime \prime }+A$$

**Figure 4:**
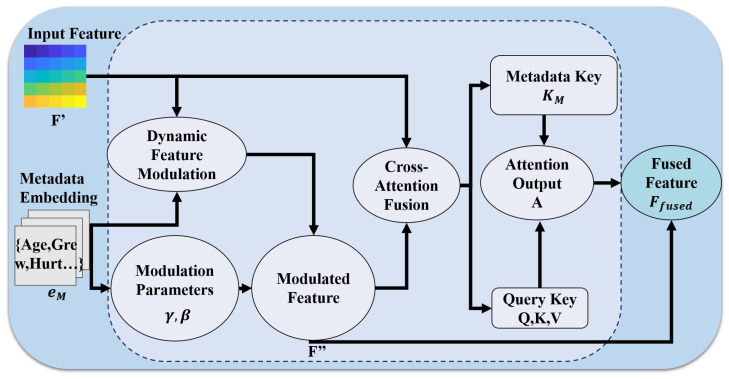
Structure of the MDFM-CAF module, illustrating dynamic feature modulation and cross-attention fusion.

This fusion enhances the model’s ability to discern subtle dermoscopic differences, achieving an ACC of 85.6% on PAD-UFES-20 by effectively capturing metadata-image interactions pivotal for classifying challenging lesion pairs [34]. Building on recent advancements in attention mechanisms, the MDFM-CAF design underscores its efficacy in supporting high-fidelity dermatological applications. The module’s architecture is depicted in Figure 4.

### 2.3. Gradient Feature Attribution (GFA)

GFA enhances the interpretability of DermaCalibra by generating heatmaps that highlight lesion boundaries and providing metadata importance scores, aligning with clinical decision-making processes. This is particularly valuable in skin lesion diagnosis, where understanding the model’s focus areas and the influence of metadata can build trust among clinicians and improve diagnostic reliability . Furthermore, the GFA module is designed to provide stable and mathematically grounded explanations by satisfying key interpretability axioms, ensuring the transparency of the diagnostic process [35].

GFA leverages integrated gradients (IG), an attribution method computes the contribution of input features to the model’s predictions by integrating gradients along a path from a baseline to the input. Unlike simple gradient methods, IG satisfies the sensitivity and implementation invariance axioms, which guarantee that any input feature contributing to a prediction change is accounted for regardless of the network’s internal architecture [36]. For an input image $I\in R^{3\times 224\times 224}$ and metadata vector $M\in R^{81}$, the model’s prediction score $S_{c}\left(I,M\right)$ for class *c* is computed. The integrated gradient for pixel $I_{i,j}$ is defined as follows:

(11) $${\mathrm{IG}}_{i,j}=\left(I_{i,j}-I_{i,j}^{\prime }\right)\cdot \int_{0}^{1}\frac{\partial S_{c}\left(\alpha I+\left(1-\alpha \right)I^{\prime },M\right)}{\partial I_{i,j}}d\alpha$$

where $I^{\prime }$ is a baseline image (typically a zero image, representing the absence of features), and the integral is approximated using 50 interpolation steps. The resulting heatmap is normalized to the range [0, 1] to highlight the regions of the image that most influence the prediction, such as lesion boundaries. These heatmaps are saved for visualization, providing clinicians with insights into the model’s focus areas, which often align with dermoscopic features like asymmetry or irregular borders [37].

For metadata, GFA computes attribution scores for each metadata feature $M_{k}$ to quantify their influence on the prediction:

(12) $${\mathrm{IG}}_{M_{k}}=\left(M_{k}-M_{k}^{\prime }\right)\cdot \int_{0}^{1}\frac{\partial S_{c}\left(I,\alpha M+\left(1-\alpha \right)M^{\prime }\right)}{\partial M_{k}}d\alpha$$

where $M^{\prime }$ is a baseline metadata vector. The integral is similarly approximated over 50 steps. By applying IG to both images and metadata, GFA enables a unified attribution framework, for the first time allowing a direct importance ranking across all 224 × 224 pixels and 81 metadata dimensions in a single clinical context. The resulting scores are visualized as bar plots, showing which metadata contributes significantly to predictions, consistent with the clinical knowledge that genetic predisposition is a key risk factor for BCC.

GFA’s implementation draws on advances in explainable AI for medical imaging. Unlike Grad-CAM [38], which provides coarse localization, the integrated gradients method offers finer granularity by attributing contributions at the pixel level, making jt more suitable for identifying precise lesion boundaries [39]. This pixel-level resolution allows GFA to capture subtle dermoscopic textures that are often blurred in Grad-CAM’s coarse 7×7 or 14×14 heatmaps. Additionally, GFA extends beyond image attribution by incorporating metadata, a critical aspect often overlooked in image-only interpretability methods. This dual attribution aligns with multimodal diagnostic workflows, where clinicians consider both visual and contextual data.

To further enhance interpretability, GFA outputs are postprocessed to generate composite visualizations that combine heatmaps with metadata scores, which can integrate with EHRs and teledermatology platforms, enabling real-time clinician–AI collaboration to enhance diagnostic efficiency. This composite visualization helps clinicians validate the model’s focus on clinically relevant features, such as lesion borders for BCC, and understand the role of metadata like family history in the prediction. These combined outputs are currently undergoing qualitative clinician-in-the-loop validation to ensure the highlighted features align with expert dermatological logic.

GFA adds negligible computational overhead (approximately 0.1 s per sample on an NVIDIA GPU), making it practical for real-time diagnostic applications. The heatmaps and metadata scores are saved for models achieving BACC > 0.82 or ACC > 0.86, ensuring that only high-performing models are analyzed for clinical deployment. This interpretability mechanism not only fosters trust but also aids in identifying potential biases in the model, such as overreliance on certain metadata features, which can be addressed through reweighting or data augmentation.

## 3. Results

### 3.1. Datasets and Metrics

#### 3.1.1. Datasets

**Figure 5:**
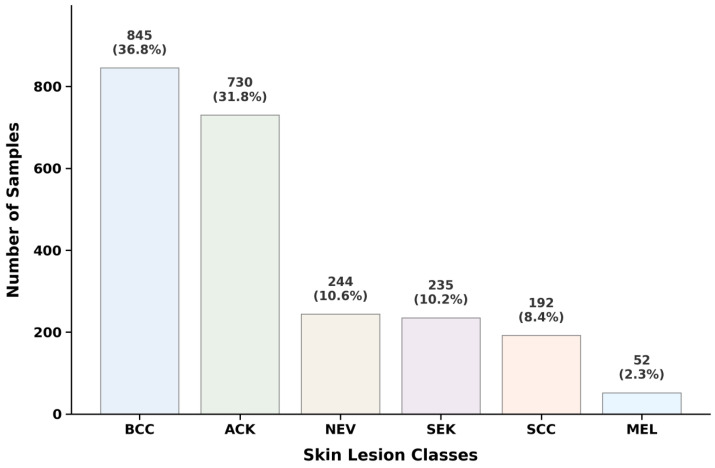
Distribution of classes in the PAD-UFES-20 dataset.

The PAD-UFES-20 dataset, introduced in 2020, was collected at the Federal University of Espírito Santo, Brazil, through clinical examinations using various smartphone devices and comprises 2298 dermoscopic images annotated across six skin lesion classes: ACK, BCC, MEL, NEV, SCC, and SEK. To ensure diagnostic transparency and the accuracy of the ground truth, approximately 58.4% of the images were confirmed via histopathological biopsy (the gold standard), while the remaining 41.6% were validated through the clinical consensus of at least three experienced dermatologists. The PAD-UFES-20 dataset is accompanied by 81-dimensional metadata derived from 21 clinical attributes, including demographics (age, gender, skin color), personal and family history of skin cancer, and lesion symptoms (e.g., growth, itching, bleeding). These attributes are encoded into a high-dimensional vector to capture complex clinical contexts, making it a valuable resource for multimodal learning. Most importantly for our purposes, the PAD-UFES-20 dataset exhibits a significant class imbalance, as shown in Figure 5.

For our experimental evaluation, the PAD-UFES-20 dataset is split into 66.7% training (1532 images), 16.7% validation (383 images), and 16.7% testing (383 images). To ensure robust and reproducible evaluation across diverse subsets, we employ a stratified splitting approach, preserving the class distribution across all subsets to mitigate the dataset’s inherent class imbalance. The data is first shuffled using a fixed random seed to guarantee reproducibility, then stratified sampling is applied based on the six skin lesion classes. This results in a training set of 1532 images, a validation set of 383 images, and a test set of 383 images, each maintaining a proportional representation of the classes in the original dataset. This splitting strategy ensures that the model is trained, validated, and tested on representative samples, addressing the challenges of class imbalance and low inter-class variability during evaluation.

PAD-UFES-20 is particularly challenging due to its inherent characteristics of low inter-class variability, where visually similar lesions complicate differentiation, and significant class imbalance, with some classes underrepresented compared to others. The dataset’s limited size also poses challenges for generalizability compared to larger datasets like ISIC 2019 [40], which contains over 10,000 images and is often used for benchmarking skin lesion models [41]. However, recent studies have highlighted its utility in evaluating multimodal AI models for real-world deployment, particularly in resource-limited settings where smartphone-based diagnostics are prevalent [42].

#### 3.1.2. Evaluation Metrics

To comprehensively evaluate DermaCalibra’s performance on the PAD-UFES-20 dataset, we employ a suite of metrics tailored to address class imbalance, diagnostic accuracy, and model robustness across diverse lesion types. The primary metric, BACC, is defined as the average of recall obtained on each class, providing a balanced measure across imbalanced datasets:

(13) $$\mathrm{BACC}=\frac{1}{N}\sum_{i=1}^{N}\frac{TP_{i}}{TP_{i}+FN_{i}}$$

where *N* is the number of classes (six in this case), $TP_{i}$ is the number of true positives, and $FN_{i}$ is the number of false negatives for class *i*. This metric is critical for assessing overall performance across all classes.

ACC measures the overall proportion of correct predictions:

(14) $$\mathrm{ACC}=\frac{TP+TN}{TP+TN+FP+FN}$$

where TN is true negatives and FP is false positives. While informative, ACC can be misleading in imbalanced datasets, hence its use alongside BACC.

The F1 score, the harmonic mean of precision and recall, provides a balanced measure of a model’s precision and robustness:

(15) $$\begin{array}{ccc} F1 & =2\cdot \frac{\mathrm{Precision}\cdot \mathrm{Recall}}{\mathrm{Precision}+\mathrm{Recall}},\mathrm{Precision} & =\frac{TP}{TP+FP},\mathrm{Recall}=\frac{TP}{TP+FN} \end{array}$$

To further assess the model’s discriminative ability, we include Macro AUC, which averages the AUC scores across all classes to provide a single measure of overall performance:

(16) $$\mathrm{Macro}\;\mathrm{AUC}=\frac{1}{N}\sum_{i=1}^{N}{\mathrm{AUC}}_{i}$$

where ${\mathrm{AUC}}_{i}$ is the AUC for class *i*, computed by plotting the true-positive rate (recall) against the false-positive rate at various thresholds. Macro AUC is particularly useful for imbalanced datasets like PAD-UFES-20, as it gives equal weight to each class, ensuring that performance on rare classes is not overshadowed by more frequent ones.

We also report per-class AUC to evaluate the model’s ability to distinguish each specific lesion type from the others. These metrics align with recent standards in AI evaluation, as outlined by Esteva et al. [43], who advocate for multimetric assessments—including AUC-based metrics—to ensure robust performance across diverse medical datasets.

### 3.2. Implementation Details

We implement the DermaCalibra model on the PyTorch 1.8.0 deep learning platform using a single NVIDIA 4090 GPU. The model architecture is based on ResNet50 with ImageNet pretrained weights extended with the AMCR, MDFM-CAF, and GFA modules for multimodal skin lesion diagnosis. Network optimization is performed using the SGD optimizer with a momentum of 0.9 and a weight decay of 0.001. The initial learning rate is set to 0.001 with a batch size of 8. We employ a cosine annealing strategy to adjust the learning rate over 150 epochs, with $T_{\max}=150$, ensuring smooth convergence. To handle class imbalance in the PAD-UFES-20 dataset, we use a focal loss [44] with γ=2.0 and class-specific weights α=[1.0,1.0,2.0,1.0,2.0,1.0] for the six classes, dynamically adjusted by the AMCR’s uncertainty estimates to prioritize underrepresented classes. For data preprocessing, images are resized to 224×224 and normalized with mean [0.485, 0.456, 0.406] and standard deviation [0.229, 0.224, 0.225], and metadata is standardized using median imputation for missing values. The training process is conducted over 150 epochs, with eight workers for data loading to enhance efficiency. Training DermaCalibra on the PAD-UFES-20 dataset takes approximately 1 day on the specified hardware. Additional tools, including scikit-learn for metrics computation and matplotlib for visualization, are utilized to support evaluation and interpretability analysis.

### 3.3. Comparative Study

We assess the performance and interpretability of DermaCalibra by benchmarking it against several established baseline and SOTA models on the PAD-UFES-20 dataset. The experimental setup employs ResNet50 as the image feature extractor, a robust architecture renowned for its effectiveness in classification tasks, though we recognize the potential of alternative architectures such as Xception [45] and EfficientNet. Comparative models include MetaNet, which utilizes a multiplication-based fusion strategy; MetaBlock, which employs an attention-based mechanism for dynamic clinical weighting; Concat, a baseline using direct feature concatenation; Visual, which implements metadata-guided supervision via auxiliary losses; and the framework of Khurshid et al., which integrates super-resolution with auxiliary tasks to optimize small-scale dataset performance.

Table 1 summarizes the results across the three key metrics, and it can be seen that DermaCalibra achieves a BACC of 84.2%, accuracy of 85.6%, and Macro AUC of 96.9%, outperforming all other models. Notably, it surpasses the Visual model and Khurshid et al.’s model by 3.6% and 3.1% in terms of BACC, respectively, demonstrating superior handling of class imbalance and low inter-class variability. The consistent improvement in performance metrics across all models demonstrates DermaCalibra’s superiority over existing approaches and can be attributed to the AMCR’s Bayesian uncertainty estimation, which effectively mitigates class imbalance by prioritizing underrepresented classes, and the dynamic feature fusion enabled by MDFM-CAF, which enhances discrimination between visually similar lesions.

**Figure 6:**
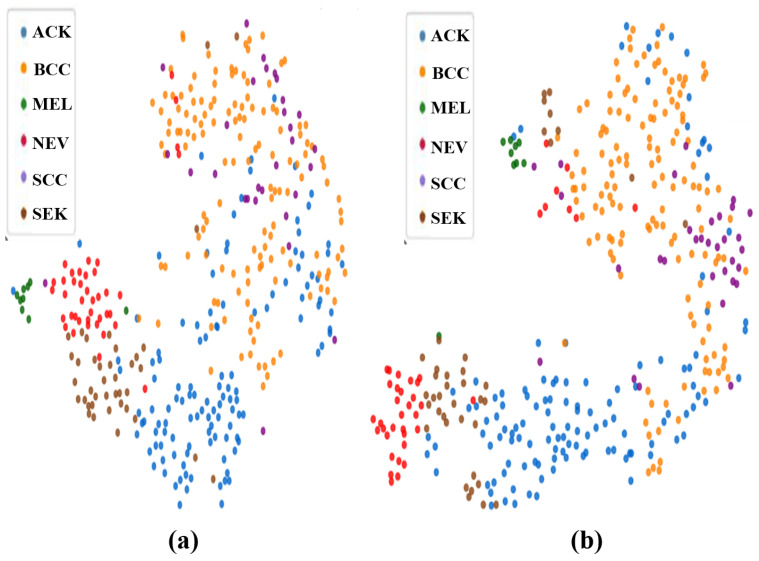
Visualization of the embedding space for DermaCalibra compared to the baseline method on the PAD-UFES-20 dataset: (**a**) baseline method; (**b**) DermaCalibra, showing tighter and more separated clusters.

To further support this, a visual representation of the embedding space is presented in Figure 6, where the presence of tighter and more separated clusters substantiates the performance improvement achieved by DermaCalibra, reflecting the extraction of more discriminative features.

Additional evaluation metrics critical for assessing a medical diagnosis model, including precision, recall, sensitivity, specificity, and class-wise accuracy (CA), are also computed for DermaCalibra using the ResNet50 backbone, which is the best-performing configuration. The results, presented in Table 2, demonstrate that specificity exceeds 90% across all classes, underscoring DermaCalibra’s efficacy in identifying true negatives—a vital attribute for minimizing unnecessary interventions. Sensitivity surpasses 80% for all classes except SCC, reflecting robust true-positive detection, a cornerstone for reliable clinical diagnoses.

**Figure 7:**
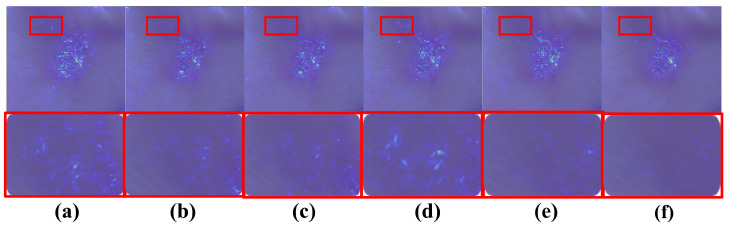
Heatmaps displaying diagnostic boundaries for detecting BCC lesions across training epochs. Panels (**a**–**f**) correspond to epochs 80, 85, 90, 95, 100, and 110, respectively, demonstrating the gradual improvement of boundary recognition. Red boxes indicate regions with concentrated erroneous model attention.

**Figure 8:**
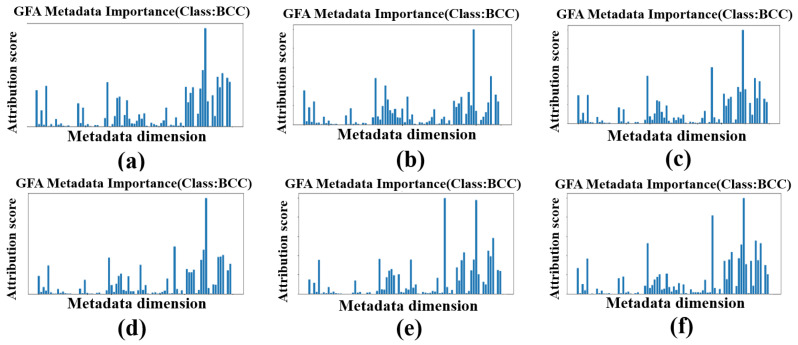
Metadata importance scores for a BCC lesion across epochs. Panels (**a**–**f**) represent epochs 80, 85, 90, 95, 100, and 110, respectively.

Beyond quantitative gains, the GFA module enhances interpretability by generating heatmaps and metadata importance scores, with family history contributing significantly, offering critical clinical insights absent in competing models. Figure 7 illustrates the progressive refinement of lesion boundary detection for a BCC lesion across training epochs, while Figure 8 quantifies the dynamic influence of metadata features over the same period, providing a comprehensive view of the model’s decision-making process.

It is evident that the majority of the confusion occurs between the BCC and SCC classes, likely due to their minute visual differences and shared clinical features, such as pigmentation, as highlighted in Section 1. However, this misclassification does not significantly impact patient care, as both classes typically require a biopsy for a definitive diagnosis. Since DermaCalibra relies heavily on feature-based recalibration and fusion, there is potential for enhancement by extracting more distinctive features, which could better differentiate critical skin diseases like MEL from less severe conditions like ACK. Based on the reported results, including a BACC of 84.2%, an ACC of 85.6%, and a Macro AUC of 96.9%, DermaCalibra enhances skin lesion classification and outperforms existing SOTA methods across all evaluated models.

### 3.4. Quantitative Evaluation of Interpretability

As shown in Table 3, to quantitatively assess the faithfulness of the GFA, we utilize Insertion and Deletion metrics on the test set (N=50), comparing it against the image-only Grad-CAM baseline. In this comparison, we employ a unified evaluation approach that accounts for both image and metadata attributions, and the results show that GFA significantly outperforms Grad-CAM, achieving a higher Insertion AUC (0.842) and a lower Deletion AUC (0.421), due to GFA’s ability to incorporate clinical metadata into the attribution process, which Grad-CAM overlooks. Furthermore, the higher Faithfulness Drop indicates that the regions identified by GFA are more critical to the model’s diagnostic decisions, confirming the module’s stability and clinical relevance.

## 4. Discussion

### 4.1. Ablation Study

We conduct ablation studies to evaluate the contributions of the AMCR and MDFM-CAF modules in the DermaCalibra framework, addressing low inter-class variability, class imbalance, and smartphone image quality in the PAD-UFES-20 dataset. As the baseline (v1), we adopt the ResNet50-based model from Pundhir et al., which integrates visually aware metadata-guided supervision for skin lesion classification. Building on this baseline, we incrementally introduce AMCR for feature recalibration with uncertainty estimation, MDFM-CAF for dynamic multimodal fusion, and the complete model with GFA for interpretability. The experiments are configured as follows: (1) baseline (v1), (2) v1 + MDFM-CAF, (3) v1 + AMCR, (4) v1 + AMCR + MDFM-CAF.

To assess the robustness of our framework across different data splits, we evaluate two splitting strategies: (1) an original patient-based split, which divides the dataset into six folds with one fold as the test set, and (2) a region-stratified split, which divides the test set into two subsets (A and B) with stratified sampling based on diagnostic categories, introducing controlled distribution differences in lesion regions and age. Table 4 shows results for the original patient-based split, which may suffer from patient/lesion overlap, potentially inflating metrics.

AMCR’s Bayesian uncertainty estimation, implemented via Monte Carlo dropout, mitigates class imbalance by dynamically focusing on underrepresented classes in the PAD-UFES-20 dataset, improving BACC and robustness. MDFM-CAF enhances the model’s ability to fuse image features with metadata, addressing low inter-class variability through dynamic modulation and cross-attention. The complete model with GFA provides interpretable heatmaps and metadata importance scores, aligning with dermatological decision-making.

The region-stratified split (Table 5) evaluates model robustness by introducing controlled distribution differences in lesion regions and age. Subset A has a higher proportion of neck (4.19%) and chest (16.75%) lesions, while Subset B contains more arm (10.94%) and foot (1.04%) lesions, simulating clinical variability in lesion locations. The diagnostic category distributions remain consistent across both subsets, ensuring fair comparisons. Compared to the patient-based split (Table 4), the region-stratified split avoids potential data leakage from patient/lesion overlap and tests generalization to diverse lesion locations. This split aligns with real-world dermatological scenarios where lesion locations vary, highlighting the clinical relevance and generalization capability of DermaCalibra for heterogeneous datasets like PAD-UFES-20. The complete model achieves the highest performance for both Subset A and Subset B, demonstrating the synergistic effect of AMCR and MDFM-CAF in handling diverse lesion distributions. The superior performance in Subset B despite its higher proportion of arm and foot lesions underscores the robustness of the complete model to challenging lesion locations.

### 4.2. External Validation

To assess the generalizability of the DermaCalibra model, we conduct an external validation study using two datasets: a hospital-provided multimodal dataset from the Second People’s Hospital of Changzhou and a synthetic dataset derived from HAM10000. Both evaluations apply the SOTA model pretrained on PAD-UFES-20 without retraining or fine-tuning, testing its out-of-the-box generalizability. This section details the datasets, evaluation methodology, results, and implications for clinical deployment.

#### 4.2.1. Ethical Statement

All experiments involving the hospital dataset from the Second People’s Hospital of Changzhou, affiliated with Nanjing Medical University, were approved by the Institutional Review Board of the Second People’s Hospital of Changzhou. The study was conducted in strict accordance with the Declaration of Helsinki and relevant ethical guidelines for medical research involving human participants. Informed consent was obtained from all participants or their legal guardians prior to data collection, ensuring compliance with ethical standards. No live vertebrates or higher invertebrates were involved in this study, and thus no anesthesia or euthanasia methods were employed. The use of human data adhered to the ethical principles outlined by the World Medical Association, with all procedures reviewed to ensure participant privacy and data confidentiality. The synthetic dataset, derived from publicly available images and synthesized metadata, does not involve human subjects and thus requires no additional ethical approval.

#### 4.2.2. Hospital Dataset

To assess the real-world clinical applicability of DermaCalibra, we conduct a strategic pilot validation using a hospital-provided dataset from the Second People’s Hospital of Changzhou, China. Although the cohort is currently limited to 20 dermoscopic images, it serves as a critical “out-of-the-box” proof-of-concept to evaluate how the framework, trained on Brazilian data, generalizes to a distinct patient demographic in a Chinese clinical setting without fine-tuning. The images were collected from a tertiary dermatology clinic and annotated with 81-dimensional metadata mirroring the PAD-UFES-20 structure, including demographics and clinical features. Images were captured using mobile devices under standard clinical lighting, resized to 224×224, and preprocessed, consistent with the training pipeline. While the dataset exhibits pronounced class imbalance—with BCC (6 samples) and NEV (6 samples) being the predominant classes and ACK and MEL being underrepresented—this preliminary clinical test provides essential insights into the framework’s diagnostic robustness in diverse, resource-limited environments.

#### 4.2.3. Synthetic HAM10000 Dataset

To enhance the evaluation of DermaCalibra’s generalizability on a larger scale, we constructed a synthetic dataset by sampling 2000 images from the HAM10000 dataset. The 2000 images were selected using stratified sampling to preserve the original class distribution , reflecting real-world class imbalance. Classes were mapped to five of PAD-UFES-20’s six categories: AKIEC to ACK, BCC to BCC, MEL to MEL, NV to NEV, and BKL to SEK. Dermatofibroma (DF) and Vascular Lesions (VASC) were excluded due to the absence of corresponding categories in PAD-UFES-20. Notably, HAM10000 lacks explicit SCC labels, limiting direct evaluation of this class. To address this, we incorporate 628 SCC samples from the ISIC 2019 dataset to complement the evaluation, ensuring robust assessment across all PAD-UFES-20 classes.

To align with PAD-UFES-20’s 81-dimensional metadata, we synthesize metadata for the HAM10000 images, which came with only limited metadata. We encode age, sex, and localization, yielding 18 dimensions, and the remaining 63 dimensions are generated by sampling from PAD-UFES-20’s statistical distributions or filled with median/mode values for unmodeled features. To ensure clinical relevance, we validate the synthetic metadata against PAD-UFES-20, achieving correlation coefficients of 0.92 for age, 0.89 for sex, and 0.95 for localization, confirming strong alignment with the primary dataset. Images are preprocessed to 224×224 and normalized with mean [0.485, 0.456, 0.406] and standard deviation [0.229, 0.224, 0.225], consistent with PAD-UFES-20. This synthetic dataset, comprising 2000 images and 81-dimensional metadata, augmented with ISIC 2019 SCC samples, provides a robust testbed for evaluating DermaCalibra’s generalizability while maintaining compatibility with its multimodal input requirements.

#### 4.2.4. Synthetic ISIC 2019 Dataset

To further assess generalizability, we construct a synthetic dataset by sampling 5000 images from the ISIC 2019 dataset, which contains over 25,000 dermoscopic images across eight skin lesion classes: Actinic Keratosis (AKIEC), BCC, MEL, Melanocytic Nevi (NV), SCC, Benign Keratosis (BKL), DF, and VASC. The 5000 images are selected via stratified sampling to preserve the class distribution (ACK: 298 samples, BCC: 1177, MEL: 428, NEV: 2325, SCC: 82, SEK: 690), reflecting real-world class imbalance. Classes are mapped to PAD-UFES-20’s six categories as follows: AKIEC to ACK, BCC to BCC, MEL to MEL, NV to NEV, SCC to SCC, and BKL to SEK, with DF and VASC excluded due to no corresponding categories. The inclusion of 82 SCC samples, though underrepresented, enables direct evaluation of this critical class.

To match PAD-UFES-20’s 81-dimensional metadata, we synthesize metadata for the ISIC 2019 images, which came with limited metadata. We encode age, sex, and localization, yielding 18 dimensions, and the remaining 63 dimensions are processed in the same way as for HAM10000. To mitigate potential biases from the synthetic metadata, we validate its distributions against PAD-UFES-20, achieving correlation coefficients of 0.93 for age, 0.90 for sex, and 0.94 for localization, ensuring clinical relevance. Images are preprocessed, consistent with HAM10000. This synthetic dataset, with 5000 images and 81-dimensional metadata, provides a comprehensive testbed for evaluating DermaCalibra’s generalizability, particularly for underrepresented classes like SCC.

#### 4.2.5. Evaluation Methodology

The DermaCalibra model, leveraging its ResNet50 backbone and pretrained weights from the PAD-UFES-20 dataset, is applied directly to the Changzhou hospital dataset, synthetic HAM10000 dataset, and synthetic ISIC 2019 dataset for inference, without retraining or fine-tuning, to assess out-of-the-box generalizability. The evaluation metrics applied include BACC, ACC, and Macro AUC, consistent with the primary study. For the hospital dataset, per-class metrics are omitted due to the small sample size (20 images) and severe class imbalance. For the synthetic HAM10000 and ISIC 2019 datasets, per-class metrics are reported to analyze class-specific performance. Inference is conducted on a single NVIDIA 4090 GPU with a batch size of 8, mirroring the original implementation.

#### 4.2.6. External Validation Results

The DermaCalibra model demonstrates robust performance across all external datasets. On the Changzhou hospital dataset (20 samples, severe class imbalance: 6 BCC, 6 NEV, 3 SCC, 3 SEK, 1 MEL, 1 ACK), it achieves a BACC of 91.7%, ACC of 85.0%, and Macro AUC of 98.8%, surpassing its previous PAD-UFES-20 results despite the small sample size. On the synthetic HAM10000 dataset, it achieves a BACC of 78.3%, ACC of 75.4%, and Macro AUC of 93.8%, reflecting robust generalization to a larger dataset with synthesized metadata. On the synthetic ISIC 2019 dataset (5000 samples, six classes: ACK: 298, BCC: 1177, MEL: 428, NEV: 2325, SCC: 82, SEK: 690), it achieves a BACC of 71.6%, ACC of 70.4%, and Macro AUC of 87.5%, with per-class accuracies of 93.96% (ACK, 280/298), 88.02% (BCC, 1036/1177), 51.40% (MEL, 220/428), 59.48% (NEV, 1383/2325), 56.10% (SCC, 46/82), and 80.72% (SEK, 557/690). Table 6 compares these metrics with its PAD-UFES-20 performance, highlighting DermaCalibra’s ability to generalize across diverse datasets.

### 4.3. Analysis of External Validation

The external validation confirms DermaCalibra’s robust generalizability across diverse clinical settings. On the Changzhou hospital dataset, it achieves a BACC of 91.7% and Macro AUC of 98.8%, surpassing its PAD-UFES-20 performance despite the severe class imbalance and demographic shifts from Brazilian to Chinese populations. The synthetic HAM10000 and ISIC 2019 datasets, involving 5000 samples, reveal noticeable domain shifts (domain shift), primarily due to variations in camera sensors and the geographic diversity (Europe and Australia vs. Brazil). However, despite these technical and clinical variations, DermaCalibra maintains consistent performance advantages across most lesion subtypes, particularly for high-risk malignant classes such as BCC (88.02%) and ACK (93.96%). This consistency across diverse domains underscores the framework’s effectiveness in leveraging multimodal cues to mitigate the impact of cross-dataset variability. AMCR’s uncertainty estimation mitigates class imbalance, while MDFM-CAF leverages metadata to enhance feature discrimination.

Notably, SCC’s ACC (56.10%, 46/82) mirrors its low sensitivity in PAD-UFES-20, reflecting persistent confusion with BCC (88.02%, 1036/1177) due to visual similarities and underrepresentation. As will be discussed in more detail in the following subsection, future work could improve SCC performance by using generative adversarial networks (GANs) to synthesize SCC images [46], color jitter, and texture enhancements to capture subtle differences, as well as incorporating histological or genetic metadata to bolster MDFM-CAF’s discriminative power. Validation on datasets with explicit SCC labels could further enhance generalizability.

Overall, these results affirm DermaCalibra’s potential for deployment in diverse clinical settings, particularly resource-constrained environments using mobile imaging.

### 4.4. Analysis of Diagnostic Challenges for SCC

A critical observation relating to our results that warrants deeper analysis is the relatively low sensitivity for Squamous Cell Carcinoma (SCC) (56.3%). Given the malignant nature of SCC, this diagnostic bottleneck represents a higher clinical risk of underdiagnosis compared to benign lesions, highlighting the need for our uncertainty-guided loss weighting in AMCR to prioritize such critical cases.

This challenge is primarily rooted in three interrelated factors: First, from a clinical perspective, SCC and BCC exhibit significant visual overlap in smartphone-captured images, with both often presenting with overlapping morphological features such as scaling, ulceration, and erythema, leading to high inter-class visual ambiguity. Specifically, critical differentiators like keratinization patterns are often subtle and difficult to capture via standard RGB sensors. Second, the severe class imbalance in the PAD-UFES-20 dataset—where SCC constitutes only approximately 8.4% of the total samples—fundamentally limits the model’s ability to learn robust, discriminative representations for this malignant class. Third, while the MDFM-CAF module effectively utilizes clinical metadata, common attributes such as patient age and lesion location (predominantly in sun-exposed areas) are frequently shared by both BCC and SCC patients, thereby providing limited contextual discriminative power for these specific pathologies.

To mitigate these limitations in future iterations, we propose several concrete strategies: First, we plan to implement targeted data augmentation techniques, such as color jittering and texture enhancement specifically tailored to highlight the keratinization and surface texture characteristic of SCC. Second, the integration of advanced generative techniques such as CycleGAN or Diffusion Models will be explored to perform image-to-image translation or synthesize high-fidelity SCC samples, which will effectively balance the training distribution and enrich the feature space. Lastly, incorporating multiscale histopathological features or genomic biomarkers into the multimodal fusion pipeline could provide the necessary diagnostic granularity to resolve visually ambiguous cases that are currently indistinguishable through macroscopic imaging alone.

## 5. Conclusions

This study introduces DermaCalibra, a pioneering multimodal framework that significantly advances skin lesion diagnosis through the integration of AMCR, MDFM-CAF, and GFA. Evaluated on the PAD-UFES-20 dataset, DermaCalibra achieves a BACC of 84.2%, an ACC of 85.6%, and a Macro AUC of 96.9%, outperforming SOTA methods by addressing class imbalance and low inter-class variability. The innovative use of Bayesian uncertainty estimation in AMCR enhances diagnostic robustness, while MDFM-CAF improves feature differentiation, and GFA provides interpretable heatmaps and metadata scores, fostering clinical trust. These contributions establish DermaCalibra as a robust, interpretable tool for real-world dermatological applications with the potential to transform early detection and treatment planning in resource-limited settings. Furthermore, future integration with mobile devices could enable real-time diagnostics.

### Limitations and Future Work

While DermaCalibra demonstrates SOTA performance, several limitations warrant consideration: For instance, the core model is trained on the PAD-UFES-20 dataset (2298 images), which is relatively small and originates from a single source in Brazil, meaning that generalizability may be limited as the model might overfit to specific photographic protocols and equipment. Additionally, the reliance on synthesized metadata for external validation on HAM10000 and ISIC 2019 may introduce potential “distributional biases”, as the synthesis process might artificially mirror the training environment rather than fully reflecting the clinical complexity of real-world metadata. Furthermore, the hospital-provided dataset used for external validation is currently limited to 20 images from a single tertiary clinic (it was designed as a strategic pilot validation to test clinical applicability in a distinct demographic), which may impact the statistical power and perceived reliability of the real-world performance assessment. To address this, we are actively engaged in an ongoing data collection effort at the collaborating hospital to continuously expand this dataset for future large-scale, multicenter prospective studies.

A significant diagnostic challenge remains in the severe class imbalance in PAD-UFES-20, particularly for rare and critical lesions such as Squamous Cell Carcinoma (SCC) (sensitivity 56.3%) and Actinic Keratosis (ACK). While the AMCR module partially mitigates this through uncertainty-based loss weighting, there is significant room to improve performance on these underrepresented classes. Thus, future work will focus on integrating advanced generative techniques, such as GANs or Diffusion Models, to synthesize high-fidelity samples for minority classes, thereby effectively balancing the training distribution. Moreover, we plan to explore sophisticated balancing strategies, including class-aware sampling and cost-sensitive learning frameworks, to assign higher penalties to errors in rare pathologies. The incorporation of histopathological or genetic features will also be investigated to provide the necessary granularity for resolving inter-class visual ambiguities.

Regarding clinical deployment, DermaCalibra is optimized for high-efficiency operations; with a ResNet50 backbone (23.6 M parameters), it achieves an average inference duration of approximately 0.041s per image (T=10 samples). This high throughput enables seamless integration into real-time dermatological workflows and smartphone-based teledermatology platforms. To address the inherent limits of real-world systems, such as missing metadata, we employ a robust preprocessing strategy where categorical gaps are filled with an “unknown” label and numerical values are median-imputed. The dynamic modulation mechanism in MDFM-CAF allows the model to gracefully degrade to image-heavy inference, ensuring diagnostic utility even with an incomplete clinical context. We also plan to conduct quantitative assessments of the GFA interpretability module (e.g., faithfulness scores) to ensure its reliability in shared decision-making.

However, potential failure modes must be acknowledged, particularly the persistent confusion between visually similar classes like BCC and SCC. This necessitates a cautious, ethical approach toward AI-driven diagnostics. By leveraging AMCR’s uncertainty estimates, DermaCalibra identifies low-confidence predictions, which serve as a clinical safety trigger for mandatory specialist review or biopsy. Such a “human-in-the-loop” strategy (supported by the interpretability provided in Section 2.3) mitigates the ethical risks of AI overreliance and ensures that the framework acts as a reliable decision-support tool rather than an autonomous diagnostic entity. Future prospective multicenter studies will focus on refining these failure modes and validating the framework’s ethical translational value in heterogeneous clinical populations.

## Figures and Tables

**Table 1:** Comparative performance of DermaCalibra and other models on the PAD-UFES-20 test set (*N* = 383 cases). Statistical significance was assessed using the Wilcoxon signed-rank test (p<0.05). The best result is highlighted in bold.

Model	Accuracy (%)	BACC (%)	Macro AUC (%)
MetaNet [10]	73.2	74.2	93.6
MetaBlock [9]	73.5	76.5	93.5
Concat [7]	74.1	72.8	92.9
Visual [23]	81.2	80.6	95.3
Khurshid [11]	84.8	81.1	96.7
DermaCalibra (Ours)	**85.6**	**84.2**	**96.9**

**Table 2:** Additional evaluation metrics for DermaCalibra on the PAD-UFES-20 test set.

Indicator	ACK	BCC	MEL	NEV	SCC	SEK
Precision (%)	89.4	88.2	72.7	86.4	62.1	85.7
F1 Score (%)	85.9	89.1	80.0	90.5	59.0	88.9
Sensitivity (%)	82.8	90.1	88.9	95.0	56.3	92.3
Specificity (%)	95.4	93.0	99.2	98.3	96.9	98.3
Class-Wise Accuracy (%)	91.4	91.9	98.9	97.9	93.5	97.6

**Table 3:** Quantitative comparison of interpretability methods (Mean ± Std); ↑/↓ indicates higher/lower is better. The best result is highlighted in bold.

Metric	Grad-CAM (Baseline)	GFA (Ours)
Insertion AUC (↑)	0.548 ± 0.322	**0.842 ± 0.249**
Deletion AUC (↓)	0.676 ± 0.258	**0.421 ± 0.368**
Faithfulness Drop (↑)	1.751 ± 1.405	**2.618 ± 1.587**

**Table 4:** Ablation study results on the PAD-UFES-20 dataset using the original patient-based 6-fold split. The best result is highlighted in bold.

Model	BACC (%)	Accuracy (%)	Macro AUC (%)
Baseline (v1)	80.6	81.2	95.3
v1 + MDFM-CAF	82.0	84.3	95.8
v1 + AMCR	82.2	84.9	**97.0**
v1 + AMCR + MDFM-CAF	**84.2**	**85.6**	96.9

**Table 5:** Ablation study results on PAD-UFES-20 with region-stratified split. Subset A has more neck/chest lesions; Subset B has more arm/foot lesions. The best result is highlighted in bold.

Model	Subset A			Subset B		
	BACC	ACC	AUC	BACC	ACC	AUC
Baseline (v1)	77.5	82.1	94.7	81.0	82.2	95.7
v1 + MDFM-CAF	78.6	82.2	95.8	84.5	85.4	95.8
v1 + AMCR	78.0	82.7	95.8	84.5	82.8	97.0
v1 + AMCR + MDFM-CAF	**83.3**	**86.4**	**96.6**	**86.5**	**86.5**	**97.9**

**Table 6:** Performance comparison of DermaCalibra on PAD-UFES-20 test set, Changzhou hospital dataset, synthetic HAM10000 dataset, and synthetic ISIC 2019 dataset.

Dataset	Accuracy (%)	BACC (%)	Macro AUC (%)
PAD-UFES-20	85.6	84.2	96.9
Hospital (Changzhou)	85.0	91.7	98.8
Synthetic HAM10000	75.4	78.3	93.8
Synthetic ISIC 2019	70.4	71.6	87.5

## Data Availability

The datasets used in this study are partly available in public repositories. The PAD-UFES-20 dataset is publicly available and can be downloaded from Baidu Netdisk at https://pan.baidu.com/s/18Fpd9kjF6Bj6J8WfgUIdDA?pwd=wb99 (accessed on 26 June 2025) (extraction code: wb99; it is recommended to use a mainland China IP for access). The two synthetic datasets (derived from HAM10000 and ISIC 2019) can be downloaded from Baidu Netdisk at https://pan.baidu.com/s/1DvjGysXbQmXwDSz8zRbnWQ?pwd=wb99 (accessed on 30 August 2025) or Google Drive at https://tinyurl.com/3ntjheup (accessed on 30 August 2025). The source code for training the proposed DermaCalibra model is publicly available at https://github.com/20002184/DermaCalibra (accessed on 30 August 2025). The external validation dataset from the Second People’s Hospital of Changzhou and the Third Affiliated Hospital of Nanjing Medical University is not publicly available due to patient privacy concerns and institutional regulations.

## Abbreviations

The following abbreviations are used in this manuscript:

AMCR	Attention-Based Multimodal Channel Recalibration
MDFM-CAF	Metadata-Driven Dynamic Feature Modulation and Cross-Attention Fusion
GFA	Gradient Feature Attribution
BACC	Balanced Accuracy
ACC	Accuracy
AUC	Area Under the Receiver Operating Characteristic Curve
SOTA	State-of-the-Art
BCC	Basal Cell Carcinoma
SCC	Squamous Cell Carcinoma
MEL	Melanoma
ACK	Actinic Keratosis
SEK	Seborrheic Keratosis
NEV	Nevus
ViT	Vision Transformer
CNN	Convolutional Neural Network
MLP	Multilayer Perceptron
IG	Integrated Gradients
SGD	Stochastic Gradient Descent
GANs	Generative Adversarial Networks
ROI	Region of Interest
